# Rapid cadmium SAD phasing at the standard wavelength (1 Å)

**DOI:** 10.1107/S2059798317006970

**Published:** 2017-06-30

**Authors:** Saravanan Panneerselvam, Esa-Pekka Kumpula, Inari Kursula, Anja Burkhardt, Alke Meents

**Affiliations:** a Photon Science, DESY, Notkestrasse 85, 22607 Hamburg, Germany; b Biocenter Oulu & Faculty of Biochemistry and Molecular Medicine, University of Oulu, Aapistie 7, 90220 Oulu, Finland; c Department of Biomedicine, University of Bergen, Jonas Lies vei 91, 5009 Bergen, Norway

**Keywords:** experimental phasing, X-ray crystallography, high-throughput crystallography, nucleotide-binding proteins, divalent cations

## Abstract

Cadmium ions can be effectively used to promote crystal growth and for experimental phasing. Here, the use of cadmium ions as a suitable anomalous scatterer at the standard wavelength of 1 Å is demonstrated. The structures of three different proteins were determined using cadmium single-wavelength anomalous dispersion (SAD) phasing. Owing to the strong anomalous signal, the structure of lysozyme could be automatically phased and built using a very low anomalous multiplicity (1.1) and low-completeness (77%) data set. Additionally, it is shown that cadmium ions can easily substitute divalent ions in ATP–divalent cation complexes. This property could be generally applied for phasing experiments of a wide range of nucleotide-binding proteins. Improvements in crystal growth and quality, good anomalous signal at standard wavelengths (*i.e.* no need to change photon energy) and rapid phasing and refinement using a single data set are benefits that should allow cadmium ions to be widely used for experimental phasing.

## Abstract 2

Single-wavelength anomalous dispersion (SAD) phasing experiments were successfully carried out at the standard wavelength of 1 Å by using cadmium ions as anomalous scatterers.

## 1. Introduction   

High-throughput crystallography projects demand rapid data collection and robust experimental phasing procedures that are suitable for various target proteins. Recent developments in sample preparation, beamline instrumentation and data-processing programs have improved the success rate in macromolecular crystallography (reviewed in Su *et al.*, 2015 ▸). The availability of microfocused X-ray beams (1–20 µm) at synchrotrons and the emergence of X-ray free-electron laser sources enable us to push the limits of crystal size required for diffraction experiments (Helliwell & Mitchell, 2015 ▸; Stellato *et al.*, 2014 ▸). However, the ‘phase problem’ still remains a hurdle in macromolecular crystallography. Experimental phasing, especially single-wavelength anomalous dispersion (SAD) phasing using either heavy atoms or naturally occurring atoms (for example S, Cl, P *etc.*), has become a fast and dominant method for macromolecular crystal structure determination owing to the advantage that one single crystal is often sufficient for successful phasing (Nagem *et al.*, 2001 ▸; Rose *et al.*, 2015 ▸). Currently, most SAD phasing experiments are routinely carried out using X-rays tuned to the absorption edge of the heavy atom being used or longer wavelengths (1.7–2.5 Å) to utilize natively occurring light elements. Heavy-atom derivatization of protein crystals normally requires incorporation *via* protein expression (for example selenomethionine labelling) or time-consuming soaking procedures. Furthermore, phasing at longer wavelengths often leads to radiation damage owing to increased absorption, beam instability and loss of high-resolution data (Wang *et al.*, 2006 ▸), and in some cases requires additional instrumentation such as a helium cone (Liebschner *et al.*, 2016 ▸) or an in-vacuum setup (Wagner *et al.*, 2016 ▸) to overcome absorption-related issues. Hence, the possibility of performing SAD phasing experiments at the standard wavelength of 1 Å would be a great advantage for high-throughput crystallography.

Metal ions play a crucial role in protein crystallization, and cadmium salts are used as a component in multiple crystallization and additive screens. Currently, there are more than 800 structures with cadmium ion as a ligand in the Protein Data Bank (PDB). Cadmium is a multivalent metal ion, with a full d_10_ orbital shell, and displays a number of different coordination abilities, including tetracoordination, pentacoordination and heptacoordination (Dokmanić *et al.*, 2008 ▸). A single surface-exposed amino acid (for example histidine, aspartate, glutamate or cysteine) is sufficient to bind a cadmium ion (Jesu Jaya Sudan & Sudandiradoss, 2012 ▸). The remaining coordination can be completed with water or other solvent molecules.

Cadmium ions promote the crystal growth of various different proteins such as ferritin, leucine/isoleucine/valine-binding protein (LIVBP; Trakhanov & Quiocho, 1995 ▸) and cardiac troponin C (cNTnC; Zhang *et al.*, 2013 ▸). In many cases, cadmium was present in the precipitant solution and forms intermolecular contacts which are essential for crystal formation. Futhermore, the addition of cadmium improves crystal morphology and diffraction quality. For example, the addition of 35–40 m*M* CdCl_2_ improved the diffraction quality of HisJ crystals considerably (Ames *et al.*, 1998 ▸). In another case, the formation of a novel cadmium cluster (CdCl_4_O_6_) proved to be important and aided in the crystal packing of the augmenter of liver regeneration protein (Florence *et al.*, 2012 ▸).

Cadmium can be a potential substitute for other divalent cations such as zinc (Meijers *et al.*, 2001 ▸), calcium (Dvir *et al.*, 2010 ▸) or magnesium (Eriksen *et al.*, 2002 ▸). In our recent work with the plant ethylene receptor (ETR1) protein, cadmium ions present in the precipitant solution replaced the Mg^2+^ ions and formed a chelation complex with adenosine diphosphate (ADP; Mayerhofer *et al.*, 2015 ▸). This substitution could be generally applicable for the experimental phasing of a wide range of nucleotide-binding proteins.

In this report, we study the capability of cadmium SAD phasing at the standard data-collection wavelength of 1 Å, at which most modern synchrotron beamlines have been optimized for beam stability and photon flux. Additionally, the formation of a cadmium–ATP complex is examined using crystals of the *Plasmodium falciparum* actin I and mouse gelsolin segment 1 complex (Vahokoski *et al.*, 2014 ▸). The *K* and *L* absorbtion edges for cadmium are at 0.46 Å and around 3.50 Å, respectively (Supplementary Fig. S1). Phasing experiments based on *L* absorption edges provide strong anomalous signal, as a large number of electrons contribute to this effect (Hendrickson, 2014 ▸). Even 8 keV above the *L* absorption edge, the anomalous contribution (*f*′′) of cadmium at a wavelength of 1 Å is still 2.3 e^−^, which is comparable to the anomalous contribution of selenium (*f*′′ = 3.4 e^−^) at its *K* absorption edge (0.97 Å). The advantage of working at this shorter wavelength is that a single data set should be sufficient for experimental phasing and model building, radiation damage is significantly reduced compared with longer wavelengths, and high-resolution data can be recorded more easily.

## 2. Materials and methods   

### 2.1. Sample preparation and crystallization   

To understand the binding of cadmium ions to proteins, three different samples were chosen. Hen egg-white lysozyme (HEWL; 129 residues) was used for its ability to bind multiple ions (Bénas *et al.*, 2014 ▸). ETR1 is a plant ethylene receptor which contains a histidine kinase domain (183 residues) and for which cadmium ions from the precipitant solution are essential for crystallization (Panneerselvam *et al.*, 2013 ▸). The *P. falciparum* actin I–gelsolin segment 1 complex (*Pf*ActI-G1; 505 residues) requires divalent-cation-coordinated adenosine triphosphate (ATP) or ADP in the actin active site, and was therefore chosen for the cadmium-substitution experiment. Crystallization details are presented in the Supporting Information. In brief, prior to crystallization, 50 mg ml^−1^ HEWL solution was incubated with 25 m*M* cadmium sulfate for 30 min at 293 K. ETR1 was crystallized in 50 m*M* cadmium sulfate, 0.1 *M* HEPES pH 7.5, 1.0 *M* sodium acetate. Prior to crystallization, the *Pf*ActI-G1 complex was treated with an excess of EGTA to remove calcium ions from the active-site ATP and was subsequently incubated with 1.5 m*M* CdCl_2_ for substitution.

### 2.2. Data collection and processing   

A single crystal was selected for each sample and X-ray diffraction data sets were collected on beamline P11 at PETRA III, DESY for HEWL and the *Pf*ActI-G1 complex and on beamline X06DA at the Swiss Light Source (SLS) for ETR1. Data-collection details are summarized in Table 1 ▸. The diffraction data sets were processed using *XDS* and scaled with *XSCALE* (Kabsch, 2010 ▸). The (*hkl*) and (−*h* −*k* −*l*) reflections were treated separately in all steps, including scaling and merging. In the integration and scaling steps, the parameters FRIDEL’S_LAW=FALSE and STRICT_ABSORPTION_CORRECTION=TRUE were used. To determine the minimum amount of data required for successful phasing and model building, a data set from a single data-collection run was divided into smaller parts and labelled according to the total rotation range. Initial SAD phasing protocols were carried out using *SHELXC*/*D*/*E* (Sheldrick, 2010 ▸) through *HKL*2*MAP* (Pape & Schneider, 2004 ▸). Further analysis was performed with a shell script using various combinations of parameters as discussed below. Heavy-atom sites were compared with their respective reference sets from the final refined model with *SITCOM* (Dall’Antonia & Schneider, 2006 ▸). For the low-resolution cutoff analysis, data sets were truncated at the scaling step with *XSCALE*. Successful *SHELXE* models were fed into *phenix.autobuild* to complete the automatic building (Adams *et al.*, 2010 ▸). Some of the low-resolution *SHELXE*-built models were further optimized with *Auto-Rickshaw* (Panjikar *et al.*, 2005 ▸) using the *MRSAD* protocol, in which several programs such as *Phaser* (McCoy *et al.*, 2007 ▸), *ARP*/*wARP* (Langer *et al.*, 2008 ▸), *RESOLVE* (Terwilliger, 2000 ▸) and *Buccaneer* (Cowtan, 2006 ▸) are used. Models were manually analyzed and built using *Coot* (Emsley & Cowtan, 2004 ▸) and final refinements were performed using *phenix.refine* (Afonine *et al.*, 2012 ▸) and the *PDB_REDO* server (Joosten *et al.*, 2012 ▸). Final refinement statistics are provided in Table 2 ▸. The coordinates and diffraction data have been deposited in the Protein Data Bank (PDB) as entries 5myy for the HEWL–cadmium complex structure and 5mvv for the *Pf*ActI-G1–CdATP complex structure. The ETR1 crystal structure was initially determined by molecular replacement and has been described elsewhere (PDB entry 4pl9; Mayerhofer *et al.*, 2015 ▸). The figures were generated using *PyMOL* v1.7.6.0 (http://www.pymol.org).

## 3. Results and discussion   

### 3.1. Presence of anomalous signal   

**Figure 1:**
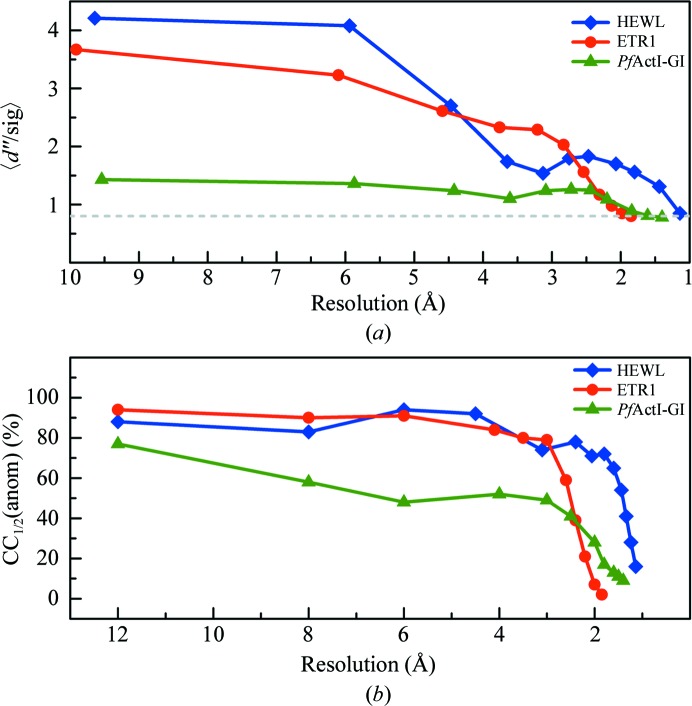
Estimation of anomalous signal. (*a*) 〈*d*′′/sig〉 plots from *SHELXC* as a function of resolution. HEWL and ETR1 show significant anomalous signal, whereas the *Pf*ActI-G1 complex crystal displays only a moderate anomalous signal up to a resolution of 1.8 Å. The dashed line (light grey) is drawn at 〈*d*′′/sig〉 = 0.8, which is considered to be zero anomalous signal. (*b*) The correlation coefficient of anomalous difference of two random half data sets [CC_1/2_(anom)] from *XSCALE* is plotted against resolution shell.

The crystals of all three different samples diffracted to high resolution. The HEWL crystal diffracted to the highest resolution of 1.1 Å. The *Pf*ActI-G1 complex and ETR1 crystals diffracted to resolutions of 1.4 and 1.85 Å, respectively. A plot of the anomalous signal indicators 〈*d*′′/sig〉 and CC_1/2_(anom) against resolution is presented in Fig. 1 ▸. The 〈*d*′′/sig〉 is calculated by *SHELXC* and the real anomalous signal above noise should be >0.8 (Sheldrick, 2010 ▸). CC_1/2_(anom), defined as the percentage of correlation between random half sets of anomalous intensity differences, is calculated with *XSCALE*. Among the three samples, HEWL and ETR1 showed strong anomalous signals up to the high-resolution limit. The *Pf*ActI-G1 complex crystal shows a weaker anomalous signal compared with the other two samples. Nevertheless, the useful anomalous signal extends to a resolution of 1.8 Å. The ratio of *R*_anom_ to *R*_p.i.m._ is also considered to be a good indicator of useful anomalous signal. All three samples showed a good *R*_anom_/*R*_p.i.m._ ratio (>2.0) which was greater than the minimum ratio of 1.5 (Mueller-Dieckmann *et al.*, 2005 ▸) recommended for experimental phasing.

### 3.2. Rapid phasing with highly redundant data sets   

In order to check the robustness of the phasing method, a rapid phasing procedure was carried out with default parameters using *SHELXC*/*D*/*E* through the program *HKL*2*MAP*. For this phasing procedure, all available data sets from the individual data-collection runs were scaled together. In all three samples, the anomalous multiplicity values were high throughout all resolution shells. The overall anomalous multiplicity values were 14.3, 6.6 and 11.3 for HEWL, ETR1 and the *Pf*ActI-G1 complex, respectively. The resolution limits for substructure determination were decided based upon the anomalous signal values in the high-resolution shells, and a conservative limit of *d*′′/sig (>0.9) was used. Since the number of heavy atoms was unknown, a default of 20 heavy atoms were searched from the three different samples, and 100 search trials were used as in simple SAD cases. The results are summarized in Table 3 ▸. In all three cases, correct substructures were determined immediately, with two well separated clusters in the plot of CC_all_ *versus* CC_weak_. The substructure solutions were ranked by their CFOM (CC_all_ + CC_weak_) values. The HEWL and ETR1 substructures had higher CFOM values (65.7 and 53.8%, respectively), whereas the *Pf*ActI-G1 complex substructure had a lower CFOM value (32.3%). Comparison of these sites with the final refined sites showed that most of the heavy-atom sites were identified and their r.m.s.d. values were lower than 1.0 Å. The substructure solutions were fed into *SHELXE* for density modification and model tracing. 20 cycles of density modification and five cycles of model tracing were chosen as default parameters. The correct hand was found quickly and model tracing resulted in a high-quality map with excellent CC values (correlation coefficient of partial structure against native data; Thorn & Sheldrick, 2013 ▸), indicating a successful tracing of the molecule. More than 80% of the residues were traced in all three samples with high correlation coefficients (HEWL, 47.24%; ETR1, 50.43%; *Pf*ActI-G1, 45.42%). Graphs from individual *SHELXC*/*D*/*E* runs are given in Supplementary Fig. S2. With such high-quality maps, *phenix.autobuild* built almost all residues automatically in the corresponding electron-density map and yielded models with low *R*_work_/*R*_free_ factors (<25%).

### 3.3. Cadmium-binding sites   

**Figure 2:**
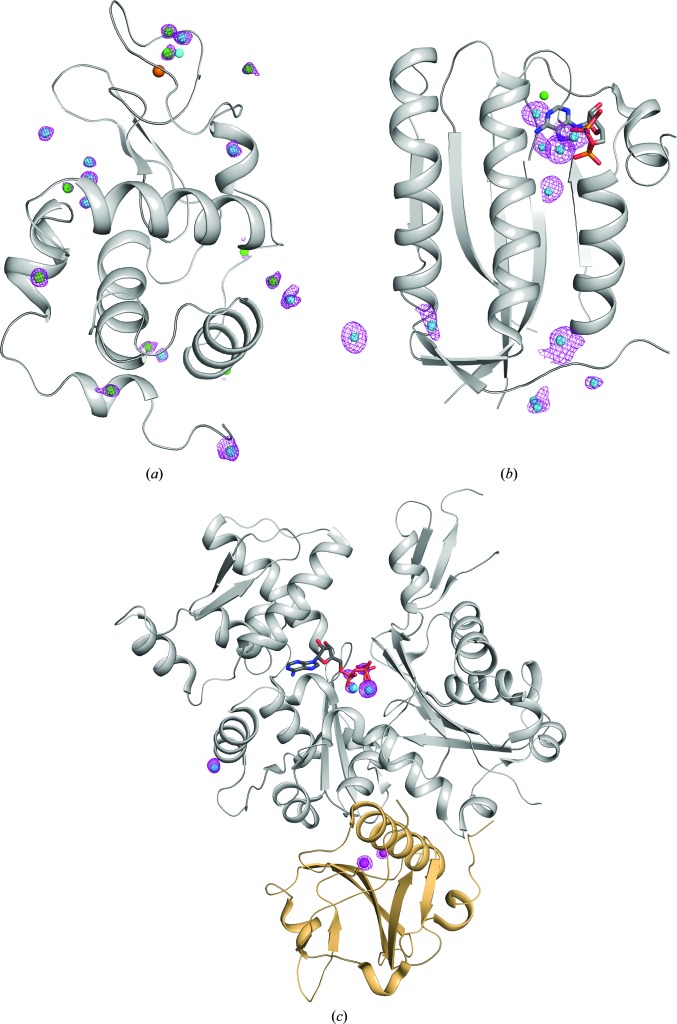
Anomalous difference Fourier maps. (*a*) HEWL, (*b*) ETR1, (*c*) *Pf*ACTI-G1 complex. The protein chains are shown as cartoons and anomalous maps are shown around ligands as purple meshes contoured at the 4σ level. In the *Pf*ACTI-G1 complex, the actin molecule is shown in grey and the gelsolin segment is shown in light orange. Cadmium ions (cyan), calcium ions (magenta), chloride ions (green) and sodium ions (orange) are shown as spheres. The bound nucleotides are shown as sticks.

Cadmium ions bind to charged residues at protein surfaces, thereby promoting crystal contacts. The cadmium-ion binding sites were initially identified based on their peak heights in the anomalous difference maps calculated with *ANODE* (Thorn & Sheldrick, 2011 ▸; Fig. 2 ▸). Since it is difficult to distinguish between different ions using a single wavelength, the binding environment was also taken into account in placing suitable ions. In this work, we observed cadmium ions with multiple coordination geometries, including tetrahedral and octahedral coordination.

#### 3.3.1. Cadmium-binding sites in HEWL   

**Figure 3:**
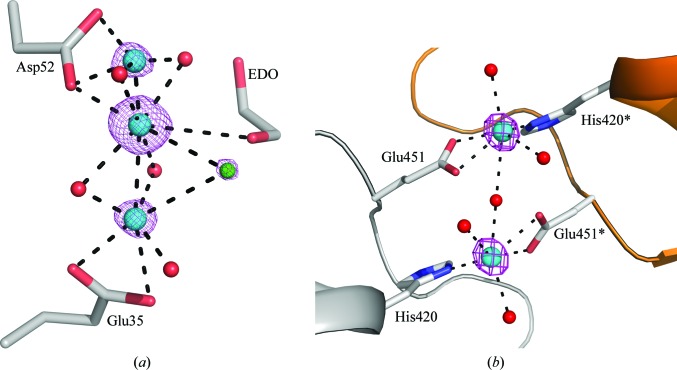
Cadmium ion-binding sites in HEWL and ETR1. (*a*) Cadmium ions binding at the active site of HEWL. A novel cadmium-chloride cluster is present in the active site and bridges the active-site residues Glu35 and Asp52. Active-site residues and ethanediol (EDO) are shown as sticks. Cadmium ions (cyan), chloride ions (green) and water molecules (red) are shown as spheres. The anomalous difference map contoured at 6σ is shown in purple. (*b*) Formation of crystal contacts by cadmium ions in ETR1. A cadmium ion (Cd605) and its symmetry equivalent bridge residues His420 and Glu451. Protein residues are shown as sticks. Parts of the protein chains are shown as cartoons in grey and orange (symmetry-related molecule). Cadmium ions (cyan) and water molecules (red) are shown as spheres. Symmetry-related residues are highlighted with an asterisk. The anomalous difference map contoured at 18σ is shown in purple.

The final model of HEWL shows no significant structural differences compared with the previously determined structures of lysozyme (PDB entry 3a8z; Ueno *et al.*, 2010 ▸). The final model contains ten cadmium ions, ten chloride ions, a sodium ion and a molecule of ethanediol, which was used as a cryoprotectant. The ions were modelled on the basis of their peak heights in the anomalous difference map (Fig. 2 ▸ *a*). Apart from the sodium ion, all others (including most of the chloride ions) gave an excellent anomalous signal. Among the modelled ions, the sodium ion and nine chloride ions were found at previously known positions (Faust *et al.*, 2008 ▸). Four chloride ions (Cl2, Cl4, Cl6 and Cl8) were coordinated to cadmium ions. Seven cadmium ions were bound to the surface of the molecule without altering the crystal lattice. The surface-bound cadmium ions are primarily coordinated to charged side chains and water molecules. Surprisingly, a novel cadmium–chloride cluster together with an ethanediol molecule was found in the active site of the enzyme (Fig. 3 ▸ *a*). Owing to partial occupancy, the exact configuration of this cluster was difficult to resolve. In this cluster, cadmium ions (Cd1, Cd2 and Cd10) form a bonding network with a chloride ion (Cl4) and the active-site residues Glu35 and Asp52.

#### 3.3.2. Cadmium-binding sites in ETR1   

A total of 11 cadmium ions and one chloride ion were modelled in the ETR1 structure based on their peak heights in the anomalous difference map (Fig. 2 ▸ *b*). Three cadmium ions interact with the ADP nucleotide, and the remaining eight cadmium ions are coordinated by charged residues on the surface of the molecule. The details of the interactions between these cadmium ions and the ADP nucleotide have been described elsewhere (Mayerhofer *et al.*, 2015 ▸). In essence, two cadmium ions (Cd601 and Cd602) are ligated to phosphates, and another cadmium ion (Cd603) is linked to N1 of the adenine ring, together with a chloride ion. Among the surface-bound cadmium ions, four (604, 605, 606 and 608) were coordinated between symmetry-related molecules, thus forming intermolecular bridges. The formation of one such crystal contact is described in Fig. 3 ▸(*b*). Cadmium ion Cd605 is present on the surface, mediating a contact between residues His420 and Glu451 from a symmetry-related molecule. It should be noted here that the presence of cadmium ions is essential for the crystallization of ETR1, as the removal of cadmium from the reservoir solution did not yield any crystals. Since ETR1 crystallizes at pH 7.5, the histidine residues (His420 and His504) are deprotonated and are therefore also involved in cadmium binding. Interestingly, His504 forms an imidazolate ion together with two cadmium ions (Cd609 and Cd610), which is rarely observed in metalloprotein structures (PDB entry 1hl5; Strange *et al.*, 2003 ▸).

#### 3.3.3. Cadmium binding and substitution of divalent metal ions in the *Pf*ActI-G1–ATP complex   

**Figure 4:**
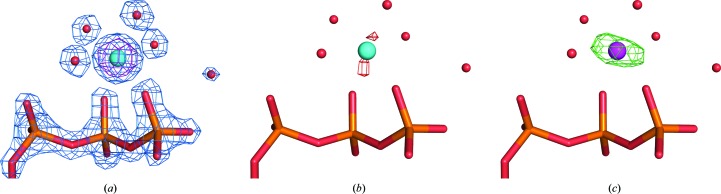
Confirmation of cadmium-ion substitution. Electron-density maps of the region surrounding the phosphate groups of ATP in the *Pf*ActI-G1 complex structure. (*a*) Placement of cadmium ion (cyan) leads to agreeable electron density and reasonable *B*-factor values. 2*F*_o_ − *F*_c_ (marine) and anomalous difference (purple) maps are shown around the cadmium ion and ATP phosphates. The 2*F*_o_ − *F*_c_ map is contoured at 3.5σ and the anomalous difference map is contoured at 18σ. (*b*) The resulting *F*_o_ − *F*_c_ map is shown as green and red meshes and contoured at 3σ. (*c*) A calcium ion (magenta) placed and refined with full occupancy led to a very low *B* factor and remaining positive density. Even at a higher σ level, a clear positive density is observed at the metal position. The *F*_o_ − *F*_c_ map is shown in green and red meshes and contoured at 5σ.

Among the three samples, the *Pf*ActI-G1 complex was crystallized with the lowest concentration of cadmium (0.75 m*M*). A total of three cadmium and two calcium ions were modelled into the *Pf*ActI-G1 complex structure based on the anomalous difference map (Fig. 2 ▸ *c*). The two calcium ions are present in the gelsolin component of the complex; they are required for complex formation (Kinosian *et al.*, 1998 ▸) and cannot be easily exchanged for other metal ions (Janmey *et al.*, 1985 ▸). Among the three cadmium ions, two (Cd1 and Cd2) are involved in coordination of the nucleotide phosphate groups, and the remaining one is bound to the surface of the molecule. Since the actin component of the complex requires ATP or ADP for stability, 1 m*M* CaATP was present during the entire purification procedure. Even though calcium ions were removed in the final step by adding an excess of EGTA (1.5 m*M*), there was a possibility of residual calcium still being bound to the ATP nucleotide. To evaluate the substitution of calcium ions by cadmium ions, the *B* factors and occupancy values of the individual ions were analyzed. The environmental *B* factors of metal-coordinating atoms were calculated with the *CheckMyMetal* server (Zheng *et al.*, 2014 ▸, 2017 ▸). When a calcium ion was placed in the active site with full occupancy, the refinement resulted in additional positive electron density and a very low *B* factor (*B*_met_ = 6.7 Å^2^) compared with the averaged *B* factor of the metal-coordination environment (*B*_env_ = 10.3 Å^2^). Placement of a cadmium ion led to a more agreeable electron density. The occupancy of this cadmium ion was then refined to 0.55 and the refined *B*-factor value (*B*_met_ = 11.1 Å^2^) was in good agreement with the average *B* factor of the environment (*B*_env_ = 10.3 Å^2^; Fig. 4 ▸). In addition, a change in the metal coordination was observed. In the native structure the active-site calcium ion typically forms a complex with a pentagonal bipyramidal geometry, in contrast to cadmium, which forms complexes with octahedral geometry. The water molecule (2813) that is present in the coordination sphere is displaced further to 4.0 Å from the metal centre (Supplementary Fig. S3). Moreover, an independent analysis was carried out with the *Phaser–MRSAD* protocol (Read & McCoy, 2011 ▸). By using the *Pf*ActI-G1 structure coordinates without cadmium ions as an initial model, the *Phaser–MRSAD* protocol unambiguously identified the cadmium ion in the correct location with an occupancy value of 0.6, which is very close to the refined occupancy value of 0.55. The log-likelihood gain values are higher upon the correct identification of this cadmium ion. These facts suggest that the bound calcium ion was indeed substituted by a cadmium ion, even at this very low concentration. The remaining two cadmium ions reside at sites that are not occupied by any ions in the native structure and were modelled based on their peak heights in the anomalous difference map. Surprisingly, one of these cadmium ions (Cd2) was coordinated by the terminal phosphate group of ATP, similarly to what was observed in the structure of ETR1.

### 3.4. Determination of the minimum necessary amount of data for phasing and model building   

Even at the absorption edge, experimental phasing generally requires high-multiplicity data to measure the inherently weak anomalous signal. If the anomalous signal can be measured accurately, then the amount of data required for experimental phasing can be significantly reduced (Wang *et al.*, 2006 ▸). In order to understand the effect of the reduction of data multiplicity and the minimum data necessary for successful phasing and model building, the data sets were successively reduced and subjected to the phasing protocol (Table 4 ▸). For HEWL and the *Pf*ActI-G1 complex, a single run was selected based on the oscillation range or sample-to-detector distance used for data collection. For HEWL, run 2, which was measured at a sample-to-detector distance of 154.6 mm, and could be recorded up to a resolution of 1.1 Å, was selected. For the *Pf*ActI-G1 complex run 3 was selected, owing to the oscillation range of 0.1°, which is roughly half the the crystal mosaicity. It has been shown that data collection with an oscillation range equal to half of the mosaicity of the crystal can substantially improve the data quality and anomalous signal (Mueller *et al.*, 2012 ▸). In the substructure-determination step, the resolution limit (SHEL) and the number of trials (NTRY) were used as variables to obtain a correct substructure solution. The density modification and model tracing were carried out with *SHELXE* using all data without any truncation.

#### 3.4.1. HEWL   

With HEWL data sets, phasing was straightforward down to a total φ rotation of 45° (Table 3 ▸ *a*). The default search parameters in *SHELXC*/*D*/*E* produced high-quality maps with excellent CC values. Upon further reduction of the data, larger numbers of search trials were required to find the correct substructure. A total φ rotation of 30° was found as the minimum that could still be used for automatic phasing and model building. Interestingly, this data set was only partially complete (77%) and had very low anomalous multiplicity (overall anamalous multiplicity value of 1.1). Using the resolution range from 50 to 1.4 Å (SHEL 50 1.4), *SHELXD* could only find four sites, which were, however, sufficient for further phase refinement and model tracing. All four sites belonged to the cluster at the active site. With additional cycles of density modification and model tracing, *SHELXE* was able to provide a high-quality map with a CC value of 32%, and a nearly complete model was traced. To our knowledge, this is the best ‘minimal’ data set used for SAD phasing experiments, in terms of completeness and multiplicity, and especially at the wavelength of 1 Å, which is far from the absorption edge of the heavy atom used.

#### 3.4.2. ETR1   

In the case of ETR1, anomalous multiplicities of 1.6, 2.4, 3.1, 4.1, 5.0 and 6.6 were observed for rotations of 90, 135, 180, 225, 270 and 360° total oscillation, respectively. Phasing and model building were successful up to a rotation range of 90° (Table 4 ▸ *b*). This data set showed an *R*_anom_/*R*_p.i.m._ ratio of 3.09 and an overall anomalous completeness of 83%. All available data were used to find heavy-atom sites using the full resolution range from 50 to 1.85 Å (SHEL 50 1.85), and three correct sites were identified with an r.m.s.d. value of 0.57 Å. Among the three sites found by *SHELXD*, two were cadmium ions (Cd601 and Cd603) complexed to ADP and the third was the cadmium ion (Cd605) bridging to the symmetry-related molecule.

#### 3.4.3. *Pf*ActI-G1 complex   

For the sequentially reduced *Pf*ActI-G1 complex data sets, SAD phasing was possible down to a total φ rotation of 120° (Table 4 ▸ *c*). All data sets showed good *R*_anom_/*R*_p.i.m._ ratios, ranging from 2.2 to 2.5. Most of the substructures had very low CFOM values (<30). Nevertheless, the correct heavy-atom sites were found with r.m.s.d. values of around 1.5 Å. For the data sets with 120 and 160° of rotation, a larger number of trials (10 000) were needed to find the correct substructure, and additional cycles of density modification and tracing were also required to obtain a good model. Unambiguous identification and location of the ATP-complexed cadmium ion was essential for all data sets.

**Figure 5:**
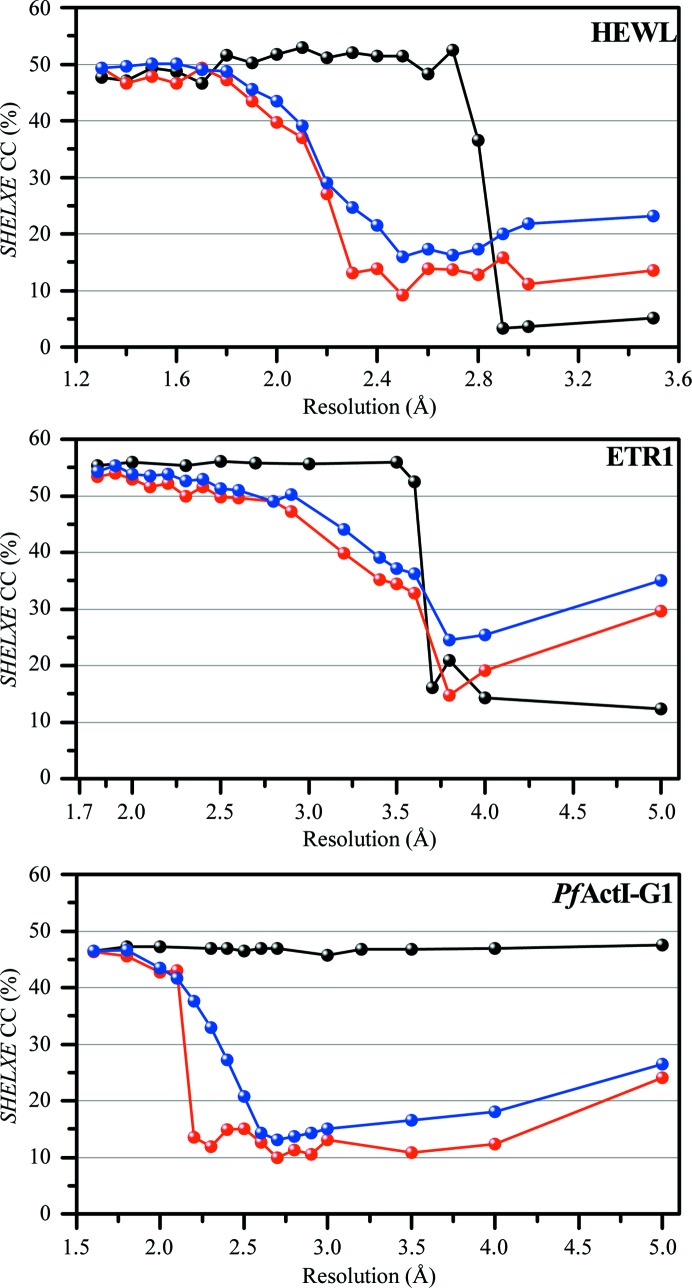
Low-resolution cutoff analysis. *SHELXE* CC values are plotted against the resolution cutoff used for the phasing procedure. The results from the resolution cutoffs at the substructure-determination step are shown in black. The results from truncated data sets are shown in red (with default *SHELXE* global cycles) and in blue (additional number of *SHELXE* global cycles).

The resolution limit played a vital role in substructure determination as well as in model building. Most of the automated phasing servers generally use lower resolution limits to locate heavy-atom sites (usually ∼0.5 Å below the high-resolution limit; Schneider & Sheldrick, 2002 ▸). In our case, SAD phasing with high-multiplicity data sets worked well at lower resolution limits, but the low-multiplicity and low-completeness data sets only worked at high-resolution cutoffs, which include all available data with usable anomalous signal. For example, with the *Pf*ActI-G1 data set with a 120° rotation *SHELXD* was only successful using a resolution limit of 1.8 Å (SHEL 50 1.8), whereas the full data set with high multiplicity could be phased even at very low resolution (5 Å; Fig. 5 ▸). Hence, we recommend testing the phasing protocol with different resolution cutoffs, especially if the data sets were collected at energies far from the absorption edge.

## 4. Phasing with low-resolution data sets   

The crystals of our three samples diffracted to high resolution. In practice, most protein crystals do not diffract as strongly, and typical data sets used for phasing are at resolutions in the range 2–3 Å. An optimal X-ray wavelength of 1.7–2.1 Å has been proposed for sulfur SAD experiments (Rose *et al.*, 2015 ▸; Mueller-Dieckmann *et al.*, 2007 ▸). Owing to the long wavelength of the incident beam, the high-resolution data cannot be recorded in most sulfur SAD experiments. In order to better understand the resolution dependence, we further tested the effect of the resolution cutoff at different steps of the phasing procedure. The resolution-cutoff analysis was carried out in two different modes. In the first mode of analysis, data sets were scaled together without any resolution cutoff and used directly for phasing. In the substructure-determination step, the desired low-resolution range was selected with the parameter SHEL. The resulting substructures were then used for *SHELXE* runs with the corresponding full resolution data sets. This mode of analysis estimates the correctness of initial substructure determination by *SHELXD* at low resolution. In the second mode of analysis, the data sets were scaled and truncated with the desired resolution limit at the scaling step. These truncated data sets were then used for the entire phasing procedure with the same resolution limit as in the scaling step. The results are illustrated in Fig. 5 ▸. The *SHELXE* CC values were used as an indicator of successful phasing (Thorn & Sheldrick, 2013 ▸).

The three samples behaved differently in the resolution-cutoff analysis. For the HEWL data set, substructure determination was possible down to a resolution of 2.8 Å. At this resolution limit, seven heavy-atom sites were found correctly, and further refinement with *SHELXE* led to the location of 16 correct heavy-atom sites. With the truncated data sets, phasing was successful to 2.3 Å resolution. For resolution limits of 2.1–2.3 Å, running additional cycles of density modification and chain tracing led to a better model with improved CC values. Using these models as a template, the *MRSAD* protocol on the *Auto-Rickshaw* web server could automatically build a nearly complete model.

In the case of ETR1, substructure determination was successful down to the resolution limit of 3.6 Å. Seven heavy-atom sites were found correctly, which was sufficient for further refinement and led to 143 residues being traced automatically with a CC value of 52.54%. To our surprise, with the truncated data sets, phasing and model building was successful down to a resolution limit of 3.6 Å. For resolution limits of 3.5 and 3.6 Å, only a partial model could be traced. At 3.5 Å resolution, 90 residues were traced by *SHELXE*, and the *MRSAD* protocol could complete the model with *R*_work_ and *R*_free_ factors of 31 and 38%, respectively. At 3.6 Å resolution, 90 residues were traced by *SHELXE*, but *MRSAD* could not improve the model. However, when the *SHELXE* output was manually inspected and trimmed to 58 residues forming well defined secondary-structure elements, this partial trimmed model could be improved further by the *MRSAD* protocol, resulting in an almost complete model (167 of 183 residues) with *R*_work_ and *R*_free_ factors of 32 and 41%, respectively.

With the *Pf*ActI-G1 complex data sets, substructure determination was possible to a very low resolution limit of 5 Å. At this resolution, all three cadmium ions and two calcium ions were correctly located, and with further phase improvement in *SHELXE* using high-resolution data 444 residues were automatically traced. In the case of the truncated data sets, phasing was possible to a resolution of up to 2.5 Å. At the resolution limit of 2.4 Å, the initial model composed of 279 residues could be improved with *MRSAD* and more than 92% of the complex (468 residues) could be built automatically.

Among the three samples, the HEWL crystal diffracted to very high resolution and exhibited a strong anomalous signal. However, the HEWL structure could only be phased down to a resolution cutoff of 2.8 Å, whereas the other two samples could be phased using data with considerably lower resolution limits (3.6 Å for ETR1 and 5.0 Å for the *Pf*ActI-G1 complex). The main differences between these three samples were the number and occupancies of heavy-atom scatterers, as well as the solvent content, which are all crucial parameters for substructure determination and density modification. Although the HEWL crystals contain more heavy atoms, their occupancies are lower (in the range of 0.2) when compared with the other samples. Owing to this and the fact that the HEWL crystals have a solvent content of only 37%, a complete model could only be built using a resolution cutoff down to 2.3 Å.

## 5. Conclusions and outlook   

High-throughput crystallography projects demand a rapid data-collection setup and robust experimental phasing of various target proteins. In this work, we showed that a single data set collected at the standard wavelength of 1 Å (12 keV) is sufficient for experimental phasing as well as final structure refinement. Cadmium ions, as used here, provide two benefits: (i) they promote crystal growth and/or improve crystal quality by mediating intermolecular bridges and (ii) their anomalous signal at the standard wavelength is very well suited for experimental phasing.

In two of our test cases, cadmium was only mixed with the protein solution and was absent from the reservoir solutions. The remaining sample (ETR1) was crystallized exclusively from a cadmium-containing precipitant solution. All three proteins crystallized in different pH regimes, suggesting that cadmium binding occurs over a wide pH range. Hence, this method can be applied to a variety of target proteins and for different crystallization conditions. The fact that cadmium can substitute the metal ion bound to a nucleotide even at very low concentrations (0.75 m*M*, as seen in the *Pf*ActI-G1 test case) suggests that it can be utilized for the generalized phasing of a wide range of nucleotide-binding and DNA-binding proteins.

Further, we showed that cadmium-based SAD phasing at the standard wavelength can be utilized for the deriving of experimental phases from a minimal data set with a very low multiplicity and completeness, as was the case for the 30° rotation data set of HEWL. This may be of particular interest for radiation-sensitive samples and for XFEL experiments, where we can expect to reduce the sample quantities as well as the beam time required for a successful structure determination.

## Supplementary Material

PDB reference: *Plasmodium falciparum* actin I–gelsolin segment 1–CdATP complex, 5mvv

PDB reference: hen egg-white lysozyme cocrystallized in the presence of cadmium sulfate, 5myy

Supporting Information.. DOI: 10.1107/S2059798317006970/di5009sup1.pdf

## Figures and Tables

**Table 1:** Data-collection parameters

	HEWL			ETR1	*Pf*ActI-G1		
Run No.	1	2	3	1	1	2	3
X-ray source	P11, PETRA			X06DA, SLS	P11, PETRA		
Wavelength (Å)	1.0332			0.998	0.9806		
Detector	PILATUS 6M			PILATUS 2M	PILATUS 6M		
Distance (mm)	368.1	154.6	800.1	180	189.3	189.3	189.3
Beam transmission (%)	5	11	11	100	21	21	21
Total No. of frames	9000	9000	9000	800	1800	1800	3600
Oscillation (°)	0.04	0.04	0.04	0.45	0.2	0.2	0.1
Exposure time (ms)	50	50	50	450	100	100	100

**Table 2:** Refinement statistics

	HEWL	*Pf*ActI-G1
PDB code	5myy	5mvv
Resolution (Å)	40–1.10 (1.13–1.10)	45–1.40 (1.42–1.40)
Total reflections	95463 (9366)	2458494 (85980)
Unique reflections	90284 (4671)	107914 (5233)
*R*_work_/*R*_free_ (%)	13.0/14.3	12.9/15.8
No. of protein residues	129	475
No. of ligand/ion atoms	21	6
Mean *B* factors (Å^2^)		
Overall	15.84	19.48
Protein	14.56	18.48
Ligands	22.57	12.34
Solvent	25.58	29.47
R.m.s.d.		
Bond lengths (Å)	0.014	0.016
Angles (°)	1.61	1.77
Ramachandran plot		
Favoured (%)	99.21	98.00
Allowed (%)	0.79	1.71
Disallowed (%)	0.00	0.19

**Table 3:** Rapid phasing results Completeness, anomalous multiplicity, 〈*I*/σ(*I*)〉 and *R*_meas_ values were calculated with *AIMLESS* (Evans & Murshudov, 2013 ▸). Values in parentheses are for the highest resolution shell. *R*_anom_ and *R*_p.i.m._ values were taken from the *SHELXC* output file (Sheldrick, 2010 ▸).

Protein	HEWL	ETR1	*Pf*ActI-G1
No. of residues per monomer	129	183	505
Space group	*P*4_3_2_1_2	*I*2_1_2_1_2_1_	*P*2_1_2_1_2
Unit-cell parameters (Å)	*a* = *b* = 78.74, *c* = 37.02	*a* = 76.32, *b* = 83.15, *c* = 91.98	*a* = 110.42, *b* = 68.92, *c* = 71.55
Solvent content	0.370	0.650	0.475
Final concentration of cadmium salt (m*M*)	12.5	25	0.75
Crystallization pH	4.6	7.5	5.9
Resolution (Å)	50–1.1 (1.12–1.10)	50–1.85 (1.89–1.85)	50–1.4 (1.50–1.40)
Completeness (%)	100 (99.85)	99.83 (99.6)	100 (99.8)
Anomalous multiplicity	14.3 (4.9)	6.6 (6.2)	11.3 (8.0)
〈*I*/σ(*I*)〉	55.1 (11.5)	32.1 (5.3)	32.0 (2.9)
*R*_meas_	0.043 (0.190)	0.045 (0.683)	0.062 (0.765)
*R*_anom_/*R*_p.i.m._ (%)	2.71 (2.57)	2.26 (1.96)	2.29 (2.24)
*SHELXD*			
Resolution (Å)	50–1.2	50–2.0	50–2.0
No. of trials	100	100	100
CFOM	65.7	53.8	32.3
CC_all_/CC_weak_	40.50/25.20	31.22/22.66	19.20/13.10
No. of sites	19/21	8/11	7/8
R.m.s.d. (Å)	0.63	1.24	0.81
*SHELXE*			
No. of residues built	122 [94%]	144 [80%]	435 [86%]
CC	47.24	50.43	45.42
*phenix.autobuild*			
No. of residues built	125 [97%]	147 [81%]	440 [88%]
*R*_work_/*R*_free_ (%)	21.55/23.14	22.45/24.20	21.8/25.04

**Table:** (*a*) HEWL.

Resolution (Å)	50–1.1 (1.12–1.1)					
Total rotation (°)	30	45	60	90	180	360
No. of frames	750	1125	1500	2250	4500	9000
Completeness (%)	77.2 (41.8)	91.7 (66.0)	95.6 (78.1)	98.3 (84.5)	99.9 (99.6)	99.9 (99.8)
Anomalous multiplicity	1.1 (1.5)	1.3 (1.3)	1.8 (1.4)	2.8 (1.8)	5.4 (2.8)	11.0 (4.9)
*SHELXD*						
Resolution limit	50–1.4	50–1.4	50–1.4	50–1.4	50–1.4	50–1.4
No. of trials	10000	10000	1000	1000	1000	1000
CFOM	14.96	25.23	23.76	41.47	53.1	63.73
CC_all_/CC_weak_	8.45/6.51	17.14/8.09	14.82/8.94	26.63/14.85	32.09/18.72	39.08/24.65
No. of sites	4	5	3	9	14	19
R.m.s.d. (Å)	1.51	2.04	0.47	0.22	0.93	0.6
*SHELXE*						
CC (%)	32.98†	37.03	41.06	45.69	46.66	50.48
No. of residues built	116† [90%]	110 [86%]	121 [93%]	120 [93%]	119 [92%]	127 [98%]

**Table:** (*b*) ETR1.

Resolution (Å)	50–1.85 (1.89–1.85)					
Total rotation (°)	90	135	180	225	270	360
No. of frames	200	300	400	500	600	800
Completeness (%)	97.4 (95.5)	99.1 (99.4)	99.8 (99.4)	99.8 (99.4)	99.9 (99.4)	100 (99.9)
Anomalous multiplicity	1.6 (1.7)	2.4 (2.3)	3.1 (2.9)	4.1 (3.8)	5.0 (4.6)	6.6 (6.2)
*SHELXD*						
Resolution limit	50–1.85	50–1.85	50–1.85	50–1.85	50–1.85	50–1.85
No. of trials	1000	1000	1000	1000	1000	1000
CFOM	26.08	38.37	34.79	42.21	44.18	53.16
CC_all_/CC_weak_	14.30/11.77	23.34/15.03	23.46/11.34	25.48/16.73	26.47/17.70	30.36/22.80
No. of sites	3	7	5	8	5	6
R.m.s.d. (Å)	0.57	1.05	0.83	1.2	1.2	0.88
*SHELXE*						
CC (%)	45.63	49.44	49.04	51.27	51.59	52.1
No. of residues built	138 [75%)	138 [76%]	140 [76%]	143 [79%]	144 [79%]	140 [76%]

**Table:** (*c*) *Pf*ActI-G1.

Resolution (Å)	50–1.40 (1.42–1.40)					
Total rotation (°)	120	160	200	240	300	360
No. of frames	1200	1600	2000	2400	3000	3600
Completeness (%)	97.3 (94.5)	99.3 (99.0)	99.9 (99.7)	99.9 (100)	99.97 (100)	99.98 (100)
Anomalous multiplicity	1.8 (1.9)	2.5 (2.2)	3.1 (2.7)	3.8 (3.2)	4.7 (3.9)	5.7 (4.7)
*SHELXD*						
Resolution limit	50–1.8	50–1.8	50–1.8	50–1.8	50–1.8	50–1.8
No. of trials	10000	10000	1000	1000	1000	1000
CFOM	15.3	16.92	16.2	21.97	22.99	26.25
CC_all_/CC_weak_	9.41/5.89	10.70/6.22	10.62/5.58	13.27/8.7	14.12/8.86	15.77/10.48
No. of sites	5	5	5	6	6	8
R.m.s.d. (Å)	1.53	0.82	0.75	0.86	1.26	0.95
*SHELXE*						
CC (%)	38.26†	36.57†	39.38	39.10	32.89	38.37
No. of residues built	435† [86%]	401† [80%]	425 [84%]	424 [84%]	373 [74%]	408 [81%]

† The low-multiplicity data sets required additional density-modification (m40) and tracing cycles (a50) compared with the default numbers (m20, a5).
