# Supplementary material for: Rapid cadmium SAD phasing at the standard wavelength (1 Å)
Source: Acta Crystallogr D Struct Biol. 2017 Jun 30;73(Pt 7):581–90. doi: 10.1107/S2059798317006970 (PMC5505155; doi:10.1107/S2059798317006970)
Supplement: Supplementary file 1 [file d-73-00581-sup1.pdf]

STRUCTURAL BIOLOGY

Volume 73 (2017)

Supporting information for article:

Rapid cadmium SAD phasing at the standard wavelength (1 Å)

Saravanan Panneerselvam, Esa-Pekka Kumpula, Inari Kursula, Anja Burkhardt and Alke Meents

S1. Protein preparation and crystallization

S1.1. HEWL

Hen egg white lysozyme was purchased from Sigma-Aldrich and used without further purification. 50 mg/ml protein solution was prepared with the 0.1 M Sodium acetate buffer (pH 4.6). To this protein solution, $CdSO_4$ was added to the final concentration of 25 mM and incubated at 295 K for 30 minutes. Crystals were obtained by the hanging-drop vapour-diffusion method at 295 K using a precipitant solution consisting of 10% (w/v) sodium chloride, 0.1 M sodium acetate (pH 4.6) and 25% (v/v) ethylene glycol. Crystals appeared and grew within few days. A single crystal of dimensions of 0.2 X 0.3 X 0.35 mm was flash-cooled at 80K in a stream of gaseous nitrogen.

S1.2. ETR1

Cloning, expression, purifcation, and crystallization of ETR1-CA domain have been described previously (Panneerselvam *et al.*, 2013). In short, the ETR1-CA protein and ADP complex was crystallized at 292K by using a precipitant solution consisting of 50 mM $CdSO_4$, 0.1 M HEPES (pH 7.5) and 1.0 M sodium acetate. Single crystals appeared with in a week. Prior to data collection, a single crystal was soaked for few seconds in a cryoprotectant solution consisting of mother liquor supplemented with 15% (v/v) glycerol.

S1.3. PfActI-G1

Plasmodium falciparum actin I (ACT1_PLAF7) and *Mus musculus* gelsolin segment 1 (residues 1-125 of A6PWS5) were purified essentially as described (Vahokoski *et al.*, 2014), with the exception that gelsolin was added to the actin preparate immediately before gel filtration in a slight excess. The gel filtration buffer composition was 10 mM HEPES pH 7.5, 0.2 mM $CaCl_2$, 1 mM TCEP, 1 mM ATP. After gel filtration, the protein was concentrated to 4.5 mg/ml, snap frozen in liquid nitrogen and stored at 193 K until use.

50 µl of the complex was thawed and 0.5 µl of 50 mM EGTA (pH 8.0) was added, resulting in a final concentration of 0.5 mM. The sample was incubated on ice for 5 min. Meanwhile, 1 µl of $CdCl_2$ was added to a fresh tube at a concentration of 15 mM. 9 µl of the incubated complex was added on the $CdCl_2$ solution, carefully mixed and filtered. Final protein concentration of the complex was 4.4 mg/ml and final concentrations of $CdCl_2$, EGTA and $CaCl_2$ were

1.5 mM, 0.45 mM and 0.18 mM, respectively. The complex was incubated on ice before crystallization for 15 min. The sample was crystallized in a mother liquor containing either 14% or 15% PEG3350 (w/v), 0.2 M KSCN and 0.1 M Bis-Tris pH 5.9. Five drops were streak-seeded from crystals of the same complex in Ca^{2+} state for each condition (14% or 15% PEG3350) after an equilibration period of 4 hours. Crystallizations were carried out at 293 K . Drop size was 0.9 µl + 0.9 µl. Well solution volume was 70 µl. The plates used were 96-well sitting drop plates (MRC). For cryoprotection, crystals were briefly soaked in 22% PEG3350 (w/v), 0.2 M KSCN, 0.1 M Bis-Tris pH 5.9 and 10% PEG400 before freezing in liquid nitrogen.

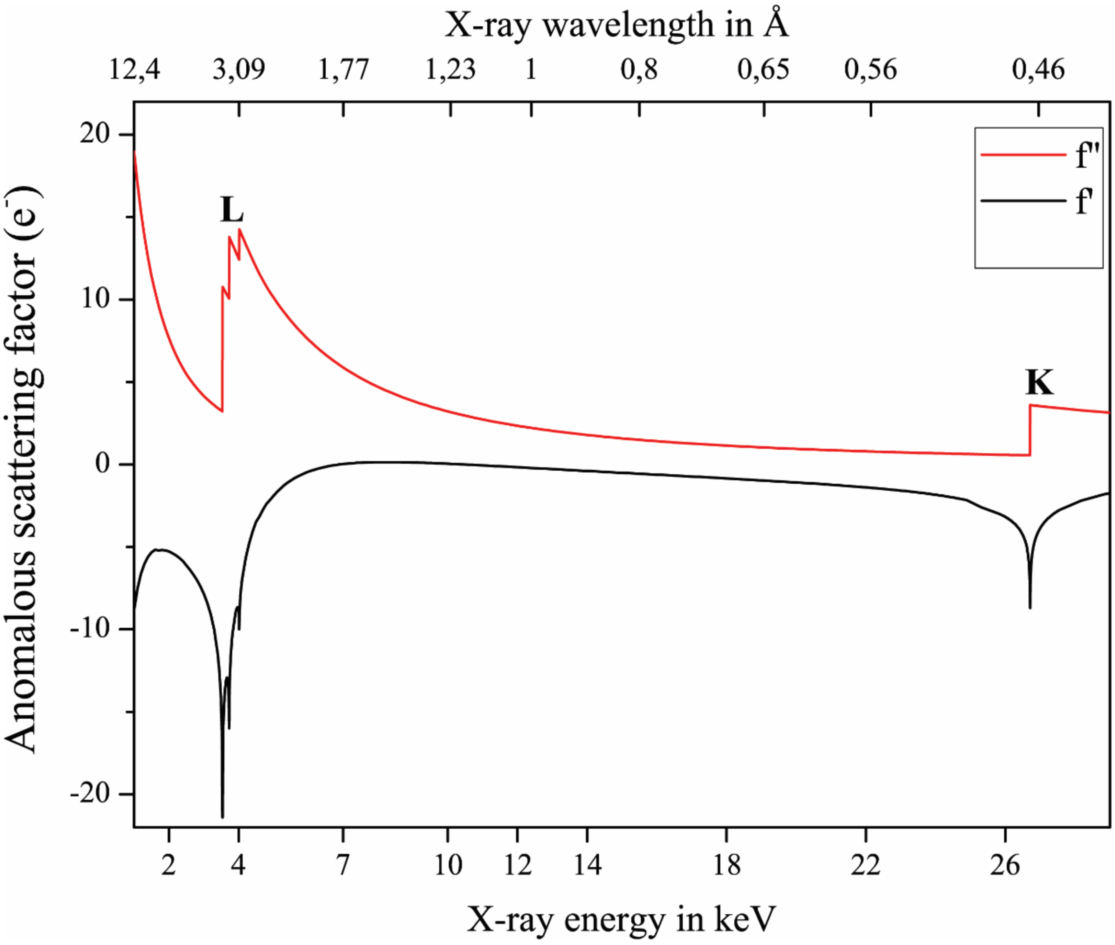

Figure S1 Anamalous scattering of cadmium. Anomalous scattering factors for cadmium for the energy range of 1 to 29 keV. L and K absoption regions are labelled. The theoritical absorption edges are L-I (4.0180 keV), L-II (3.7270), L-III (3.5375) and K (26.7112). Anomalous scattering factors were obtained from http://www.sasakiken.net/scatfac/scatfac.html

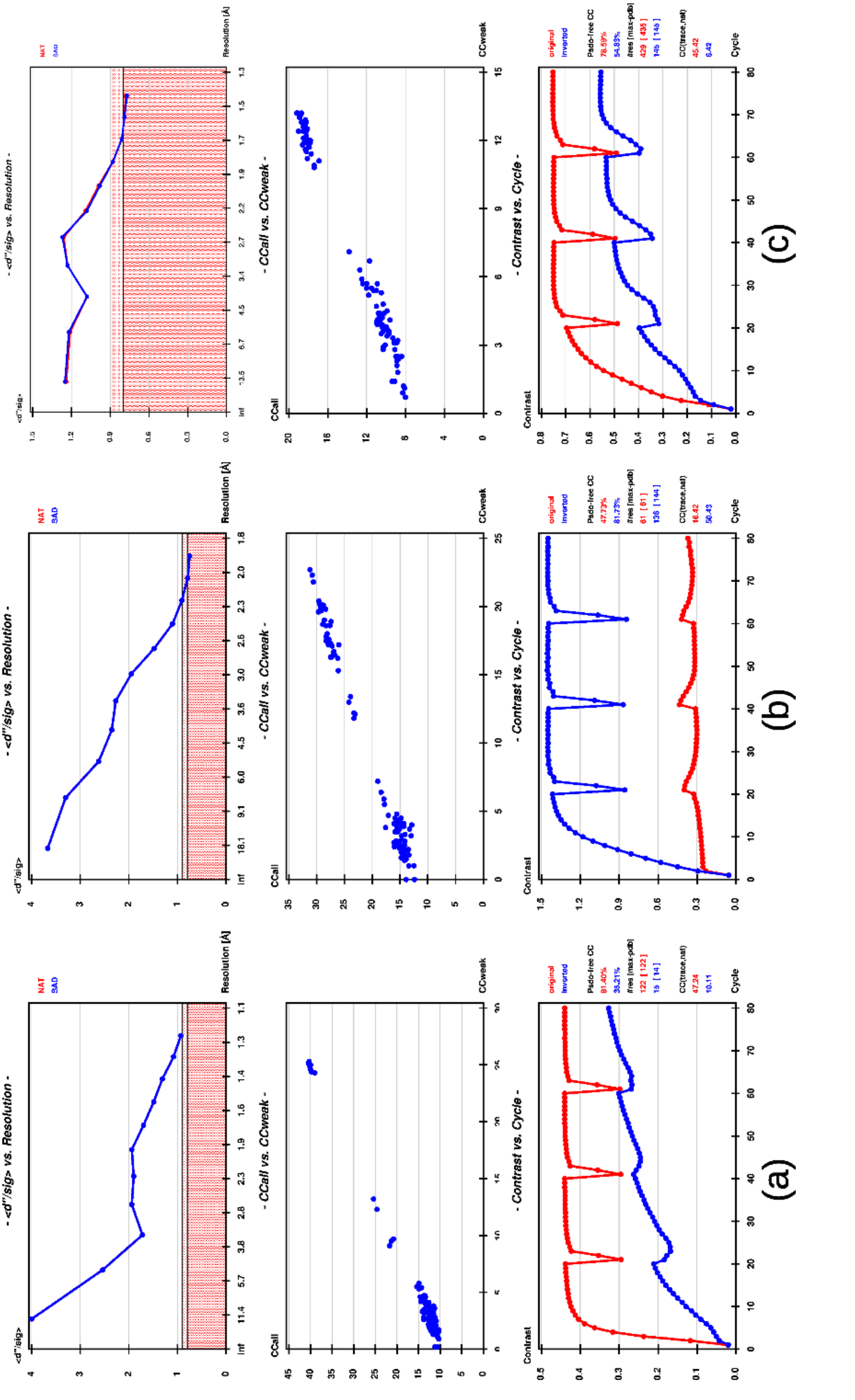
- <d''/sig> vs. Resolution -
- CCall vs. CCweak -
- Contrast vs. Cycle -
NAT
SAD
Resolution [Å]
CCall
CCweak
Contrast
Cycle
original
inverted
(a)
(b)
(c)

Figure S2 Rapid SAD phasing plots from *SHELX-CDE.* (a) HEWL, (b) ETR1, and (c) *pf*ACTI-G1 complex. The <d"/sig> plot from *SHELXC* show the presence of anomlaous signal. CC_{all} vs CC_{weak} plot from *SHELXD* run of 100 phase trials, show a clear distinct cluster of results. Contrast vs Cycle plot from *SHELXE* show the selection of correct hand and model quality.

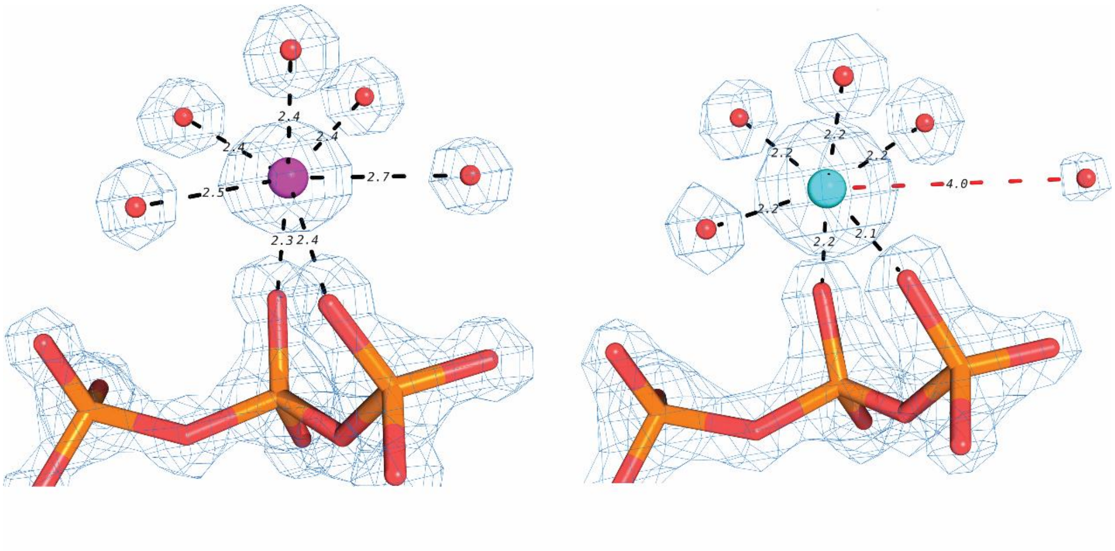

(a) (b)

Figure S3 Changes in the metal coordination. (a) In native structure (PDB ID: 4CBU), calcium ion which bound to ATP phosphates, attains a pentagonal bipyramidal coordination. (b) In the cadmium ion bound structure (PDB ID 5MVV), the cadmium ion bound to ATP forms an octahedral geometry. The coordinated water molecule (2010 in native structure, 2813 in cadmium bound structure) is displaced from the distance of 2.7 Å to 4.0 Å. $2F_o$-F_c electron density maps (marine) contoured at 2σ is shown around the metal ion and ATP nucleotide phosphates. The calcium ion (magenta), cadmium ion (cyan) and water molecules (red) are shown as spheres.

References

Panneerselvam, S., Kaljunen, H. & Mueller-Dieckmann, J. (2013). *Acta Crystallogr. Sect. F Struct. Biol. Cryst. Commun.* **69**, 1307–1309.

Vahokoski, J., Bhargav, S. P., Desfosses, A., Andreadaki, M., Kumpula, E. P., Martinez, S. M., Ignatev, A., Lepper, S., Frischknecht, F., Sidén-Kiamos, I., Sachse, C. & Kursula, I. (2014). *PLoS Pathog.* **10**, e1004091.
